# TIM‐3 and CEACAM1 do not interact in *cis* and in *trans*

**DOI:** 10.1002/eji.201948400

**Published:** 2020-04-28

**Authors:** Annika De Sousa Linhares, Florian Kellner, Sabrina Jutz, Gerhard J. Zlabinger, Hans‐Joachim Gabius, Johannes B. Huppa, Judith Leitner, Peter Steinberger

**Affiliations:** ^1^ Division of Immune Receptors and T Cell Activation Center for Pathophysiology Infectiology, and Immunology Institute of Immunology Medical University of Vienna Vienna Austria; ^2^ Institute for Hygiene and Applied Immunology Center for Pathophysiology Infectiology and Immunology Medical University of Vienna Vienna Austria; ^3^ Division of Clinical and Experimental Immunology Center for Pathophysiology, Infectiology, and Immunology Institute of Immunology Medical University of Vienna Vienna Austria; ^4^ Faculty of Veterinary Medicine Institute for Physiological Chemistry Ludwig‐Maximilians‐University Munich Germany

**Keywords:** CEACAM1, Coinhibition, Costimulation, T‐cell activation, TIM‐3

## Abstract

TIM‐3 has been considered as a target in cancer immunotherapy. In T cells, inhibitory as well as activating functions have been ascribed to this molecule. Its role may therefore depend on the state of T cells and on the presence of interaction partners capable to perform functional pairing. Carcinoembryonic antigen‐related cell adhesion molecule (CEACAM1) has been proposed to bind TIM‐3 and to regulate its function. Using a T cell reporter platform we confirmed CEACAM1‐mediated inhibition, but CEACAM1 did not functionally engage TIM‐3. TIM‐3 and CEACAM1 coexpression was limited to a small subset of activated T cells. Moreover, results obtained in extensive binding studies were not in support of an interaction between TIM‐3 and CEACAM1. Cytoplasmic sequences derived from TIM‐3 induced inhibitory signaling in our human T cell reporter system.

Our results indicate that TIM‐3 functions are independent of CEACAM1 and that this receptor has the capability to promote inhibitory signaling pathways in human T cells.

## Graphical abstract

The role of TIM‐3 and CEACAM1 was studied in a T cell reporter system. CEACAM1–CEACAM1 interaction coinhibited T cell activation, whereas CEACAM1 did not functionally engage TIM‐3 in *cis* and in *trans*. A chimeric receptor system revealed inhibitory signaling via the cytoplasmic tail of TIM‐3

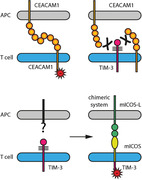

## Introduction

TIM‐3 belongs to the T cell Ig and mucin domain family of receptors and was originally considered as a marker for Th1 helper cells involved in the regulation of macrophage activation. Subsequent studies suggested a role of TIM‐3 as an inhibitory molecule on T cells mediating downregulation of the Th1 cell response and maintenance of Treg function. TIM‐3 is upregulated on CD4^+^ and CD8^+^ T cells upon activation and repeated stimulation. Additionally, TIM‐3 was described as a marker for exhausted T cells [1, 2, 3, 4]. Consequently, this glycoprotein is often assigned to a group of inhibitory T cell‐expressed receptors such as PD‐1, CTLA‐4, and LAG‐3, which are also upregulated upon repeated stimulation with antigen‐related and have been associated with a state of exhaustion in T cells [5]. Results obtained with antibodies targeting CTLA‐4 and PD‐1 pathways have impressively demonstrated that blocking such receptors with antibodies, so‐called immune checkpoint inhibitors, can be a powerful strategy to combat certain types of cancer. Several clinical trials are currently evaluating the capacity of antibodies targeting additional immune checkpoints. Among them, there are studies testing TIM‐3 alone or in combination with PD‐1 antibodies (https://clinicaltrials.gov).

However, the role of TIM‐3 in immunity is complex and incompletely understood. This receptor is also broadly expressed on cells of the innate immune system [6, 7]. Several lines of evidence suggest that TIM‐3 can function as an activating receptor on these cells [8, 9]. Moreover, several reports demonstrated that TIM‐3 can also have an activating role in T cells [10, 11, 12, 13, 14].

The cytoplasmic domain of TIM‐3 harbors several tyrosine residues but they are not part of classical motifs such as ITIMs, Immunoreceptor tyrosine‐based switch motif (ITSM), or ITAMs, which confer activating or inhibitory properties to noncatalytic tyrosine‐phosphorylated receptors [15]. However, in mouse TIM‐3, phosphorylation at Y256 and Y263 were reported to mediate interactions with SH‐2 domain containing signaling enzymes, including Lck, ZAP‐70, SLP‐76, p85 of PI3K, and PLC‐γ [12, 14]. BAT‐3/BAG6 was described to associate with the cytoplasmic domain of TIM‐3 and to repress inhibitory signaling via this receptor [16].

Several molecules have been reported to bind TIM‐3 and have been implicated in mediating TIM‐3 function. Consistent with a prominent role in innate immune cells, TIM‐3 has been reported to bind phosphatidylserine and high mobility group box 1 [17]. Galectin‐9 (Gal‐9), a tandem repeat type β‐galactoside‐binding lectin, has been proposed to serve as a binding partner for TIM‐3 and to mediate apoptosis of TIM‐3^+^ Th1 cells via functional pairing [18]. We have performed a series of binding studies and functional experiments that did not yield evidence for a specific interaction between TIM‐3 and Gal‐9 [19]. Several other molecules including 4‐1BB, CD40, CD44, and dectin‐1 were also described as counterreceptors for Gal‐9 [20, 21, 22, 23]. Of note, the functional pairing of galectins with their counterreceptors depends on the presence of suited glycosylation, a structural parameter that can vary with cell type and state of activation [24, 25, 26]. Gal‐9 was shown to exert inhibitory functions and to induce apoptosis independently of TIM‐3, and it was found that functional TIM‐3 antibodies do not block Gal‐9 function [23, 27].

Recently, carcinoembryonic antigen‐related cell adhesion molecule 1 (CEACAM1), also known as CD66a, was reported as an interaction partner for TIM‐3 that is critically involved in TIM‐3‐mediated tolerance and exhaustion. The N‐terminal V‐type Ig‐like domains of TIM‐3 and CEACAM1, which are highly similar, were responsible for this interaction. According to Huang et al., CEACAM1 promoted surface expression of TIM‐3 and these two receptors were extensively coexpressed on human and mouse T cells [28]. CEACAM1 is an activation‐induced T cell‐expressed receptor that induces inhibitory signaling and also marks exhausted T cells [29]. This receptor is a self‐ligand that is present as a homodimer. Homophilic interaction in *trans* was also described for this receptor [30]. More than ten CEACAM1‐isoforms generated by alternative splicing have been reported, but T cells predominantly express variants harboring four Ig‐like domains and a long cytoplasmic domain containing inhibitory motifs (ITIMs) [29].

In this study, we have analyzed coexpression of CEACAM1 and TIM‐3 on human T cells during activation. In addition, we have used a T cell reporter platform to test the impact of TIM‐3 on CEACAM1 function. Finally, a series of assays were performed to test the capability of TIM‐3 to interact with CEACAM1. Our study did not provide evidence for an interaction of TIM‐3 and CEACAM1 neither in *cis* nor in *trans*. In addition, we showed that these two receptors do not modulate each other´s function. Furthermore, our results indicate that the cytoplasmic tail of TIM‐3 can mediate inhibitory signaling in T cells.

## Results

### Human TIM‐3 is constitutively expressed on human myeloid cells and upregulated on T cells upon stimulation

**Figure 1:**
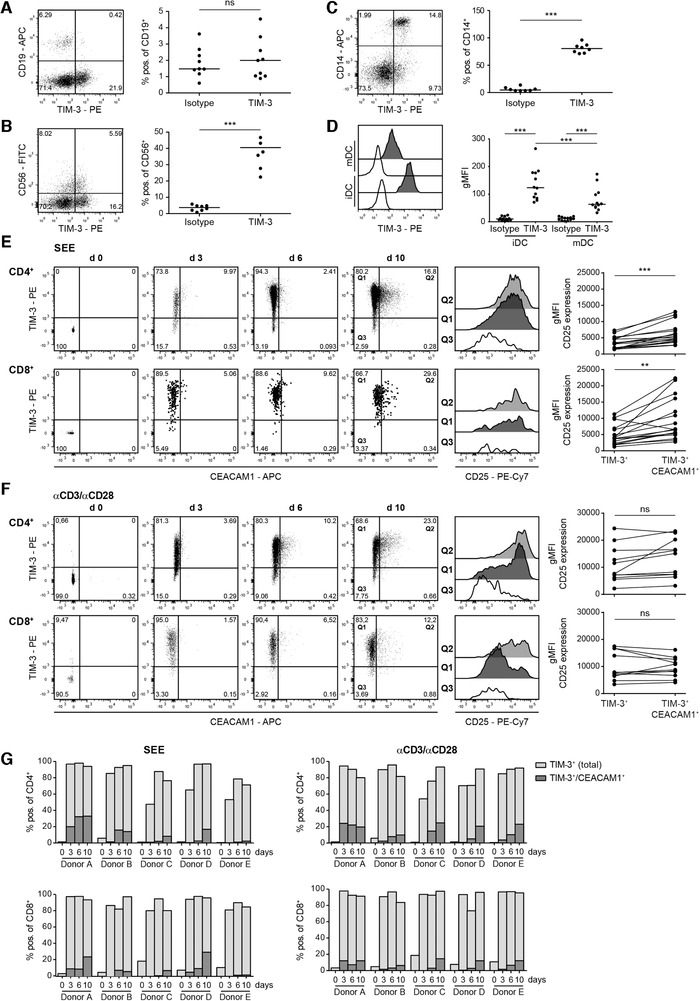
Expression analysis of TIM‐3 and CEACAM1. Flow cytometry analysis of TIM‐3 expression on freshly isolated PBMCs derived from healthy donors. CD19^+^ cells (A), CD56^+^ cells (B), and CD14^+^ cells (C) were analyzed for TIM‐3 expression. (D) Immature (iDC) and mature DCs were stained with TIM‐3 mAb (black histograms) or isotype control (open histograms). (A–D) Left: one representative donor; right: each dot represents one donor. Median is shown. (E and F) CD4^+^ and CD8^+^ T cells in freshly isolated PBMCs and PBMCs stimulated in vitro with staphylococcal enterotoxin E (SEE) (E) or immobilized aCD3/aCD28 mAb (F) for 3, 6, and 10 days were analyzed for TIM‐3, CEACAM1, and CD25 expression, respectively. In the stimulated samples, the gate was set on proliferated (CFSE^low^) cells. Left panels: dot plots from one representative donor are shown. For better visibility larger dots were used in dot plots depicting TIM‐3 and CEACAM1 expression in CD8^+^ T cells upon stimulation with SEE. Middle‐right panels: histogram overlay shows gMFI of CD25 expression on day 10 of the indicated populations. Right panels: summarized data of CD25 expression in the TIM3^+^ versus TIM3^+^/CD66a^+^ subset upon SEE (17 donors/8 experiments with 1–3 donors each) or aCD3/aCD28‐stimulation (11 donors/6 experiments with 1–3 donors each). (G) Bar diagrams from five representative donors (from three experiments with one or two donors) show percentages of CD66a^+^‐expressing cells within the TIM3^+^ population. For statistical evaluation, paired *t*‐tests (A–C; E and F) and one‐way ANOVA followed by Tukey's multiple comparison test (D) were performed (***p ≤ 0.001; **p ≤ 0.01; *p ≤ 0.05; ns, *p* > 0.05).

Analysis of TIM‐3 on freshly isolated PBMCs of healthy donors revealed that TIM‐3 is barely expressed on human B cells, whereas about 40% of CD56^+^ cells were TIM‐3^+^ (Fig. 1A and B). Monocytes as well as human monocyte‐derived DCs stained uniformly for presence of TIM‐3, indicating that human myeloid cells are marked by constitutive TIM‐3 expression (Fig. 1C and D). Interestingly, upon LPS‐mediated maturation of monocyte‐derived DCs a significant downregulation of TIM‐3 was detected, whereas PD‐L1 was upregulated (Fig. 1D; Supporting information Fig. 1A). TIM‐3 and CEACAM1 (CD66a) have been described to be coexpressed on T cells and the inhibitory function of TIM‐3 has been reported to be mediated by CEACAM1 [28]. T cells freshly isolated from healthy donors were negative for TIM‐3 and CEACAM1 (Fig. 1E). Upon in vitro stimulation with the superantigen staphylococcal enterotoxin E (SEE), we observed homogeneous upregulation of TIM‐3 on proliferating CD4^+^ and CD8^+^ T cells, and both subsets remained TIM‐3^+^ in the course of the experiment. In contrast, CEACAM1 upregulation was delayed and restricted to a subset of proliferating CD4^+^ and CD8^+^ T cells. CD25 expression was higher on TIM‐3/CEACAM1 double‐positive CD4^+^ and CD8^+^ T cells, indicating that these cells have a higher state of activation than TIM‐3^+^ CEACAM1^−^ T cells (Fig. 1E). Similar results were obtained with CD4^+^ and CD8^+^ T cells activated with CD3/CD28 antibodies (Fig. 1F). The gating strategy for in vitro activated CD4^+^ and CD8^+^ T cells is laid out in Supporting information Fig. 1B. Experiments with five donors demonstrated high percentages of TIM‐3 single‐positive and low percentages of TIM‐3/CEACAM1 double‐positive CD4^+^ and CD8^+^ T cells upon stimulation with SEE and CD3/CD28 antibodies (Fig. 1G).

### Evaluation of CEACAM1 inhibitory function in a reporter cell system

**Figure 2:**
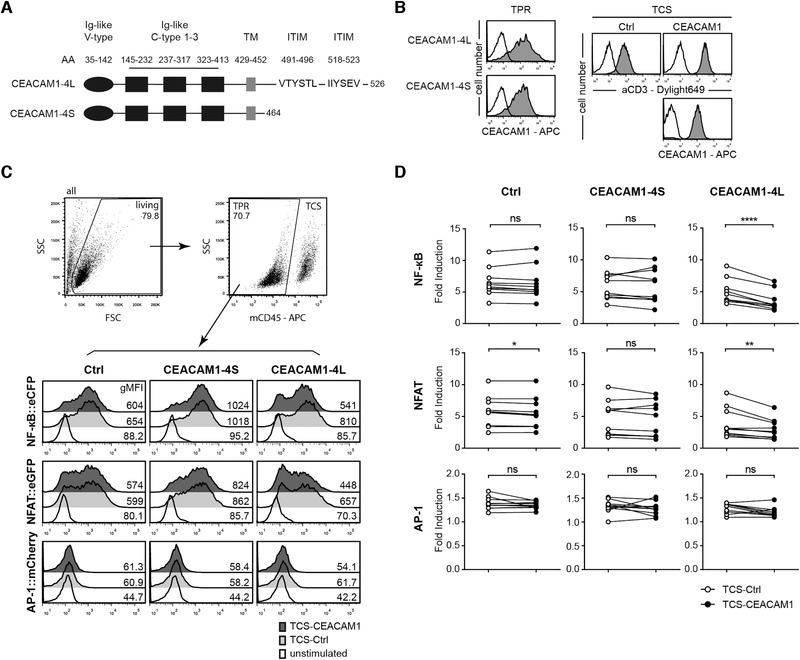
Evaluation of CEACAM1 function in a reporter cell system. (A) Schematic of CEACAM1‐4L and CEACAM1‐4S proteins. (B) Flow cytometry analysis of triple parameter reporter cells (TPR) and T cell stimulator cells (TCS). Open histograms: control cells; filled histograms: expression of indicated molecules on TPR and TCS. (C) Gating strategy and one representative stimulation experiment of control TPR and TPR‐expressing CEACAM1‐4S and CEACAM1‐4L with control TCS or TCS‐expressing CEACAM1 is shown. eGFP, eCFP, and mCherry expression was measured via flow cytometry. The histograms of unstimulated cells are also depicted. The geometric MFI (gMFI) value is shown for each histogram. (D) The indicated TPRs were stimulated with control TCS or TCS‐expressing CEACAM1. Reporter activation is shown as fold induction (gMFI of TCS‐stimulated cells/gMFI of unstimulated cells). Results are from ten independent experiments performed in triplicates. Note that some data points overlap. For statistical evaluation, paired *t*‐tests were performed (****p ≤ 0.0001; **p ≤ 0.01; *p ≤ 0.05; ns, *p* > 0.05).

CEACAM1 has been described as a heterophilic ligand for TIM‐3, which endows TIM‐3 with inhibitory functionality [28]. In primary T cells, the expression of these molecules marks subsets that are characterized by distinct activation and differentiation states, which renders the functional consequences of TIM‐3 and CEACAM1 difficult to assess. Here, we have used a previously described triple parameter reporter (TPR) system based on the human Jurkat T cell line to assess how TIM‐3 and CEACAM1 may influence each other on a functional level [31]. With these cells, the activity of transcription factors that play major roles in T cell activation processes, that is, NF‐κB, NFAT, and AP‐1, can be measured independently and simultaneously via the expression levels of the fluorescent proteins eCFP, eGFP, and mCherry, respectively. Importantly, our reporter cells can be used for gain‐of‐function studies for both receptors, since they are devoid of TIM‐3 and CEACAM1 expression. For CEACAM1, many isoforms generated by alternative splicing have been described [29]. Here, we focused on two major isoforms expressed in T cells, namely CEACAM1‐4L and CEACAM1‐4S. Human CEACAM1‐4L has one Ig‐like V‐type and three Ig‐like C2‐type domains in its extracellular domain and harbors a long cytoplasmic tail containing two ITIM motifs. The extracellular and transmembrane part of CEACAM1‐4S is identical to that of CEACAM1‐4L, but this isoform lacks most of the intracellular domain, including the ITIM motifs (Fig. 2A). Homotypic binding has been demonstrated for the Ig‐like V‐type region of CEACAM1, which mediates *cis* and *trans* interactions of this receptor [29, 32]. To evaluate CEACAM1 in our TPR system, reporter cells expressing CEACAM1‐4L or CEACAM1‐4S were generated (Fig. 2B). Reporter cells can be stimulated with T cell stimulator cells (TCS), which have a membrane‐bound anti‐CD3 Ab fragment and thus activate the T cell reporter cells by triggering their TCR‐CD3‐complex. TCS‐expressing CEACAM1 were generated to evaluate the effect of homophilic in *trans* CEACAM1 interaction during the activation of the T cell reporter cells (Fig. 2B). Coculture experiments of TPR‐CEACAM1‐4L and TPR‐CEACAM1‐4S with control‐TCS or with TCS‐expressing CEACAM1 were performed. Stimulation of TPR‐expressing CEACAM1‐4L with control TCS induced similar reporter activation as in TPR‐expressing CEACAM1‐4S or control TPR (Fig. 2C), indicating that the expression of CEACAM1‐4L per se did not exert significant inhibitory effects in the T cell reporter cells. In contrast, we observed significantly reduced activation of NF‐κB and NFAT when TPR‐CEACAM1‐4L were stimulated with TCS‐expressing CEACAM1 (Fig. 2C and D), indicating that engagement of CEACAM1‐4L in *trans* induced inhibitory signaling. Similar reporter activity in control‐TPR and CEACAM1‐4S‐TPR was induced, when these cells were stimulated with control‐TCS or CEACAM1‐TCS (Fig. 2C and D). These results show that our T‐cell reporter platform can be used to investigate inhibitory signaling of CEACAM1‐4L. Moreover, our data suggest that homophilic engagement of CEACAM1‐4L must occur in *trans* to induce effective inhibition via this receptor.

### CEACAM1 does not modulate TIM‐3 function

**Figure 3:**
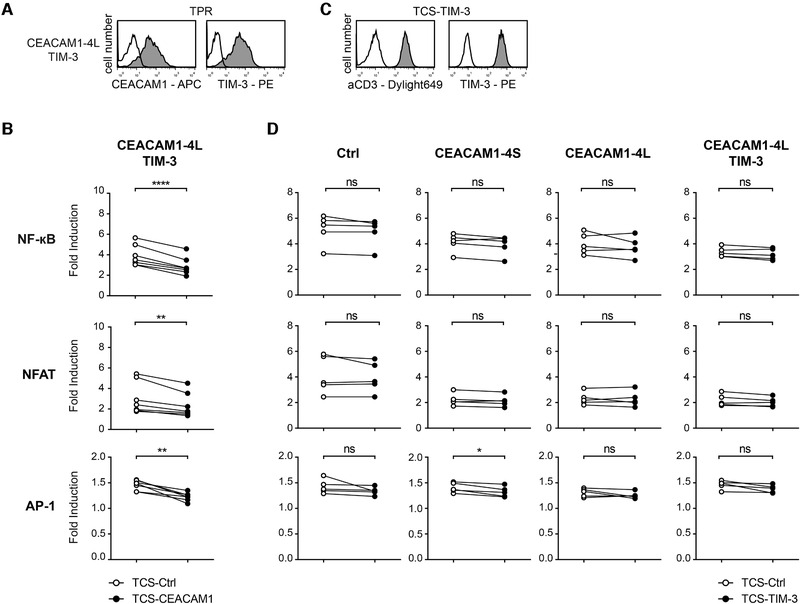
TIM‐3 does not modulate CEACAM1 function. (A) Flow cytometry analysis of TPR reporter cells coexpressing CEACAM1‐4L and TIM‐3 (gray histograms). Reactivity of antibodies to CEACAM1 and TIM‐3 to control TPR is shown as open histograms. (B) CEACAM1‐4L reporter cells coexpressing TIM‐3 were stimulated with control TCS or TCS‐expressing CEACAM1. Results are from seven independent experiments performed in triplicates. (C) Expression of membrane‐bound anti‐CD3 Ab fragment and TIM‐3 on TCS‐TIM‐3 (gray histograms). Reactivity of the used antibodies to control cells is shown as open histograms. (D) The indicated TPRs were stimulated with control TCS or TCS‐expressing TIM‐3. Results are from five independent experiments performed in triplicates. (B and D) eGFP, eCFP, and mCherry expression was measured via flow cytometry. Reporter activation is shown as fold induction (gMFI of TCS‐stimulated cells/gMFI of unstimulated cells). Note that some data points overlap. For statistical evaluation, paired *t*‐tests were performed (****p ≤ 0.0001; **p ≤ 0.01; ns, *p* > 0.05).

To assess whether the presence of TIM‐3 has effects on CEACAM1 in *cis*, TPR coexpressing CEACAM1‐4L/TIM‐3 were generated (Fig. 3A). They were then stimulated with control TCS or TCS‐CEACAM1. We observed a lower level of activation upon stimulation with TCS‐CEACAM1 also with these reporter cells (Fig. 3B), indicating that the presence of TIM‐3 did not affect inhibitory signaling via CEACAM1‐4L. Since TIM‐3 has been reported to bind CEACAM1 in *trans* also via its V‐type Ig domain [28], we generated TCS‐expressing TIM‐3 to analyze whether these cells would, like TCS‐CEACAM1, also induce reduced activation in TPR‐expressing CEACAM1‐4L as shown in Fig. 3C. However, the expression of TIM‐3 on TCS did not alter their stimulatory capability regardless of the presence of CEACAM1‐4L on the reporter cells (Fig. 3D). This indicates that TIM‐3 cannot functionally engage CEACAM1 in *trans*.

**Figure 4:**
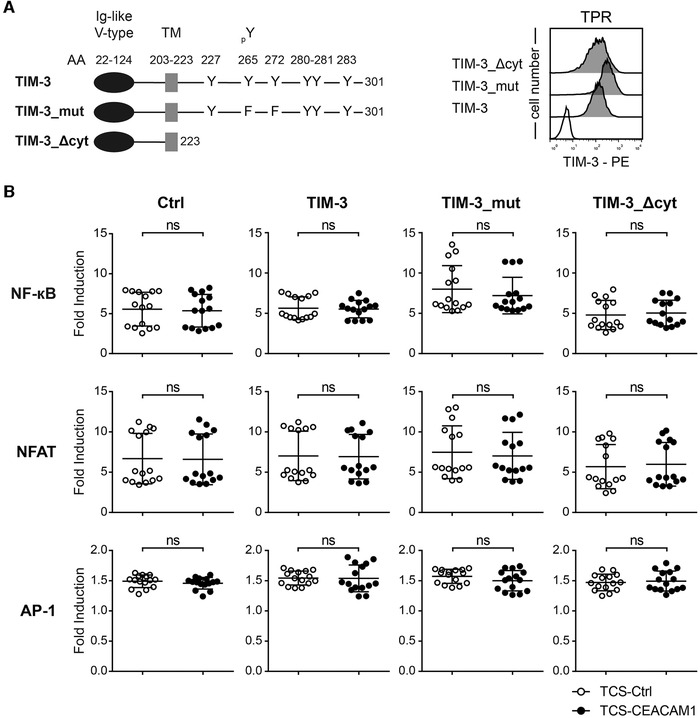
CEACAM1 does not mediate TIM‐3 signaling. (A) Schematic of WT and mutated TIM‐3 molecules (left) and flow cytometric analysis of TPR reporter cells expressing the indicated TIM‐3 molecules (gray histograms) and control TPR (open histogram). (B) The indicated TPR were stimulated with control TCS or TCS‐expressing CEACAM1. eGFP, eCFP, and mCherry expression was measured via flow cytometry. Results are shown for five independent experiments performed in triplicates. Reporter activation is shown as fold induction (gMFI of TCS‐stimulated cells/gMFI of unstimulated cells). For statistical evaluation, unpaired *t*‐tests were performed (ns, *p* > 0.05).

We also assessed whether the activation of TIM‐3‐expressing T cell reporter cells would be affected by the presence of CEACAM1 on TCS. For this, we generated TPR cells expressing full‐length TIM‐3 and, for control purposes, also a mutated variant lacking the tyrosine residues previously implicated in TIM‐3 signaling (Y265F; Y272F; TIM‐3_mut [14]) and a truncated TIM‐3 lacking the entire cytoplasmic domain (TIM‐3_Δcyt; Fig. 4A). Although previous reports found that the expression of TIM‐3 in Jurkat cells strongly modulated their function by enhancing or inhibiting TCR‐complex induced signaling [14, 33, 34], we did not observe a significant impact of the presence of TIM‐3 on our Jurkat T cell reporter cells (Fig. 4B; Supporting information Fig. 2 and data not shown). We tested TIM‐3‐reactive antibodies previously shown to enhance T cell responses [1, 4, 35, 36] on TIM‐3‐expressing reporter cells. In these experiments, we did not observe enhanced reporter activation in the presence of TIM‐3 antibodies (Supporting information Fig. 2). Importantly, the extent of stimulation of TIM‐3‐expressing reporter cells was not affected by the presence of CEACAM1 in *trans*, since control TCS and TCS‐expressing CEACAM1 induced similar reporter activation (Fig. 4B). Taken together, our data indicate that TIM‐3 did not modulate CEACAM1 function and CEACAM1 is not capable to functionally engage TIM‐3.

### TIM‐3 and CEACAM1 do not interact in *trans*

**Figure 5:**
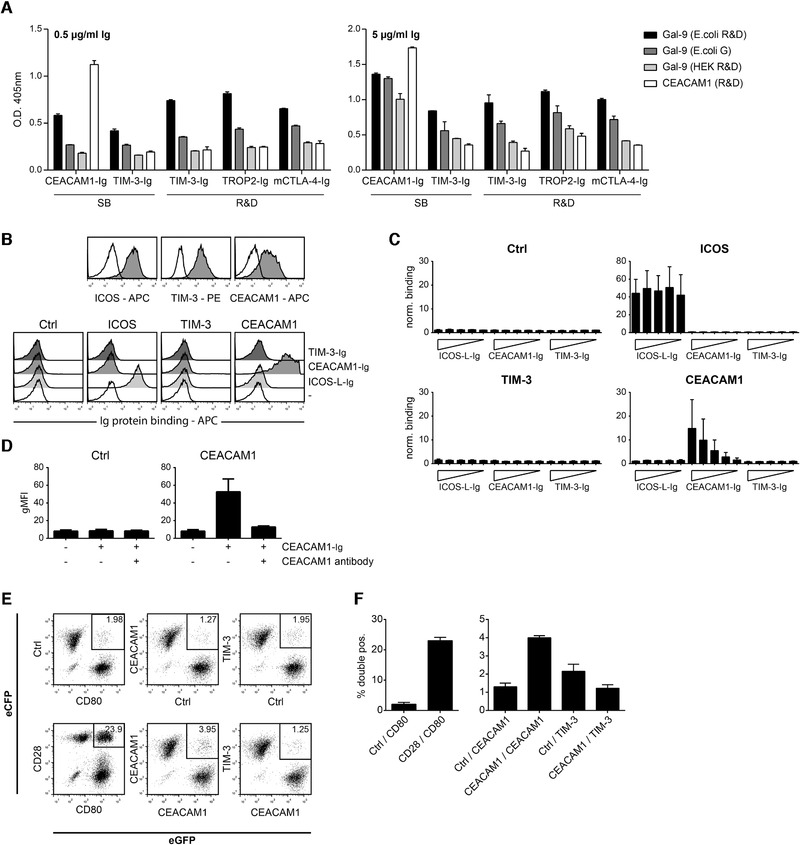
TIM‐3 and CEACAM1 do not interact in *trans*. (A) Binding of indicated Ig fusion proteins to immobilized recombinant galectin‐9 (Gal‐9) and CEACAM1 proteins was analyzed by ELISA. Results are representative for four independently performed experiments. Information on the used proteins is provided in the material and method section. (B) Jurkat cells transduced to express ICOS, TIM‐3, or CEACAM1 were probed with the respective antibodies and analyzed by flow cytometry (gray histograms). Open histograms show the reactivity of control Jurkat cells. Bottom: results of a representative binding experiment of indicated Ig fusion proteins used at a final concentration of 10 μg/mL to control Jurkat cells or Jurkat cells expressing ICOS, TIM‐3, or CEACAM1. Binding was detected via flow cytometry by an APC‐conjugated goat‐anti‐human IgG (Fc‐specific) Ab. (C) Binding of indicated Ig fusion proteins (final concentrations: 31.6, 10, 3.16, 1, and 0.316 μg/mL) to control Jurkat cells and Jurkat cells expressing ICOS, TIM‐3, or CEACAM1. Binding was detected as described in (B). Results are shown from three different experiments performed in duplicates. Binding signals (gMFI) were normalized to background binding (gMFI values obtained with secondary reagent only). (D) Binding of CEACAM1‐Ig (final concentration: 10 μg/mL) to control Jurkat cells and CEACAM1‐expressing Jurkat cells in absence or presence of a blocking CEACAM1 mAb. Results are shown from four different experiments performed in duplicates. (E) Cells coexpressing eCFP or eGFP and the indicated cell surface molecules were preincubated for 1 h and conjugate formation between cells expressing different fluorescent proteins was assessed via flow cytometry. (F) Results of three independently performed cell–cell binding assays are summarized. (A, C, D, and F) Standard deviation is shown.

To assess interactions between TIM‐3 and CEACAM1, we performed a series of binding assays. First, we tested the interaction of Ig fusion proteins representing TIM‐3 (TIM‐3‐Ig) with immobilized CEACAM1 in an ELISA. We used TIM‐3 fusion proteins from two sources, a fusion protein representing the extracellular domain of CEACAM1 (CEACAM1‐Ig) served as positive control. TROP2‐Ig and mCTLA‐4‐Ig were used as negative controls. Gal‐9, which has also been reported as a binding partner for TIM‐3, was also tested in these experiments. Most functional data with Gal‐9 were generated with bacterially expressed proteins because recombinant Gal‐9 from a eukaryotic source was not commercially available until recently. In our binding experiments, we tested material of three recombinant human Gal‐9 preparations, two expressed in *E. coli* and one expressed in the human embryonic kidney (HEK) 293T cell line. Representative binding experiments are depicted in Fig. 5A. Both TIM‐3 fusion proteins failed to bind immobilized CEACAM1, while this molecule was strongly bound by CEACAM1‐Ig, confirming a homotypic interaction of CEACAM1. Bacterially expressed Gal‐9 bound all fusion proteins. The strongest binding signals were observed with CEACAM1‐Ig and TROP2‐Ig. In line with previous results obtained in our laboratory, these ELISA‐based results did not point to a specific interaction between TIM‐3 and Gal‐9. Interestingly, compared to both bacterially expressed Gal‐9 preparations, binding of Gal‐9 expressed in mammalian cells to all Ig fusion proteins was significantly weaker (Fig. 5A).

In the next set of experiments, we tested the interaction of membrane‐resident TIM‐3 and CEACAM1 molecules with Ig fusion proteins representing TIM‐3 and CEACAM1. Jurkat E6.1 cells expressing either human TIM‐3 or human CEACAM1 were generated and expression of homogeneous levels of these molecules was confirmed by flow cytometry (Fig. 5B, top). Jurkat E6.1 cells expressing ICOS and ICOS‐L‐Ig served as an additional positive control in these experiments. We could observe binding of CEACAM1‐Ig to CEACAM1‐expressing cells in a dose‐dependent manner. Importantly, neither binding of TIM‐3‐Ig to CEACAM1‐expressing cells nor binding of CEACAM1‐Ig to TIM‐3‐expressing cells was observed (Fig. 5B bottom and C). Specific interaction of CEACAM1‐Ig with CEACAM1 could be confirmed using a blocking Ab (Fig. 5D).

Binding studies with recombinant proteins representing the extracellular domains of receptors depend on the integrity of the protein preparations, and, in addition, experiments with soluble proteins might not always reliably reflect the interaction of membrane‐resident molecules, which is influenced by the biophysical characteristics of cell membranes and the topological constellations of membrane‐resident receptors. To investigate receptor–ligand interaction in *trans* under more physiological conditions, Xiao et al. have recently described an elegant cell conjugation assay, where one type of transfected cell was labeled with a red dye, whereas the other transfected cell was labeled with a green dye [37]. The binding of the two cells could be determined by measuring double‐positive cell conjugates by flow cytometry. We have adopted and modified this assay to evaluate a potential interaction of TIM‐3 and CEACAM1 in *trans*. Instead of employing transfected cells labeled with a fluorescent dye, we used cells stably expressing the fluorescent proteins eGFP and eCFP. A high percentage of double‐positive cells could be detected for CD28‐eCFP with CD80‐eGFP, which were used as a positive control (Fig. 5E). We could also confirm the homotypic interaction of CEACAM1 in this assay. However, compared to cells expressing CD28 and CD80 the number of eGFP/eCFP double‐positive cell conjugates was considerably lower. By contrast, we could not detect significant binding of TIM‐3‐expressing cells to CEACAM1^+^ cells (Fig. 5E and F). Thus, fully in line with the functional results, our binding data do not provide evidence for an in *trans* interaction between human CEACAM1 and TIM‐3.

### TIM‐3 and CEACAM1 do not interact in *cis*

**Figure 6:**
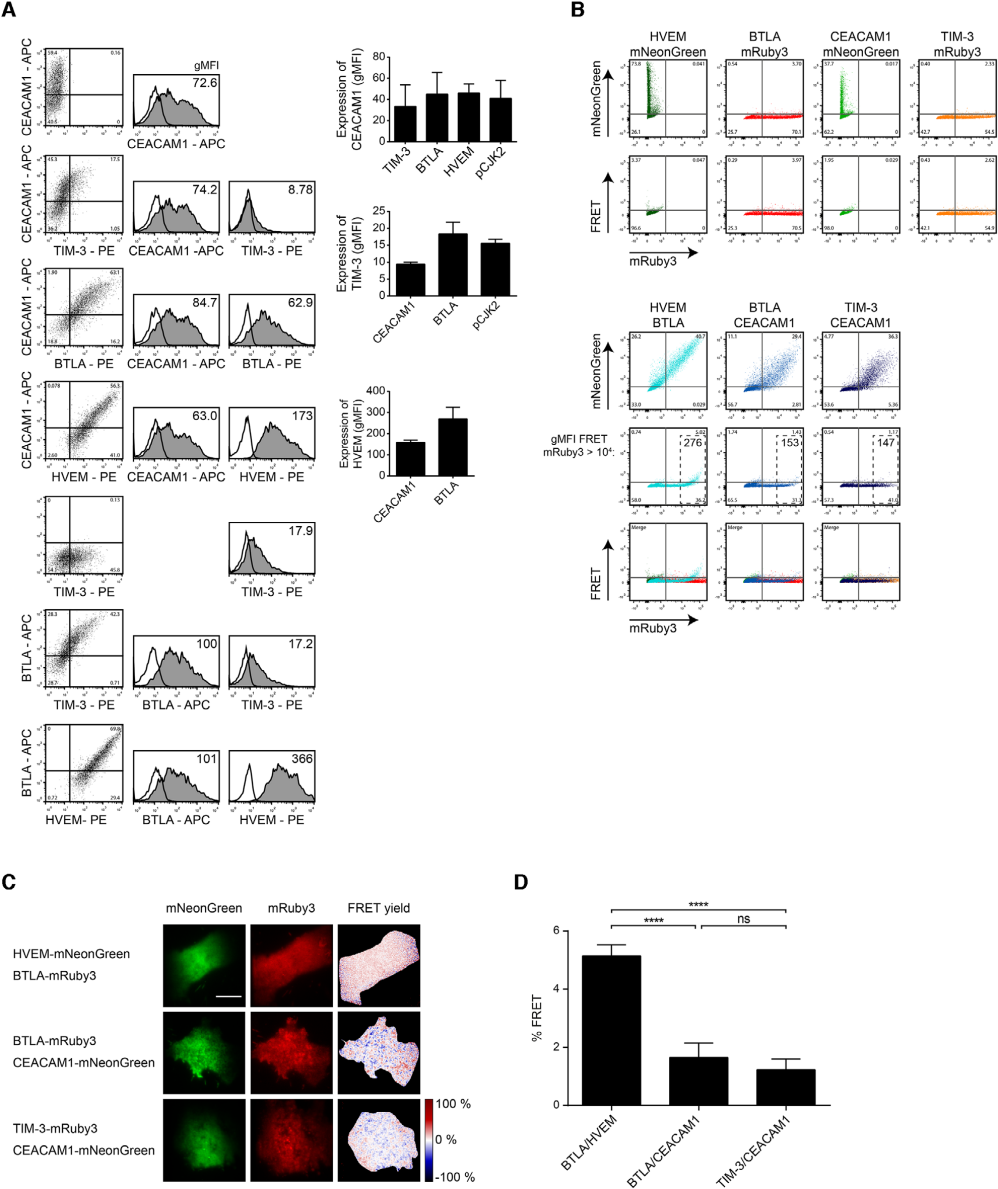
TIM‐3 and CEACAM1 do not interact in *cis*. (A) HEK293T cells were cotransfected with indicated molecules. Flow cytometry analysis was performed 2 days after transfection. Open histograms: staining with an isotype control; filled histograms: staining of indicated molecules. Bar diagrams show results of three independent experiments. (B) Analysis of in *cis* association of indicated interaction partners via FRET. mRuby3 (561 nm excitation; PE filter) and mNeonGreen (488 nm excitation; FITC filter) fusion constructs were cotransfected in HEK293T cells and analyzed by flow cytometry. For FRET detection, 488 nm laser light was used for fluorophore excitation, and mRuby3 emission (600 nm longpass filter) was measured. (C) Fluorescence images of HEK293T cells coexpressing the indicated molecules. FRET yield was determined by donor recovery after acceptor photobleaching and calculated pixelwise. Representative images are shown. Scale bar = 5 μm. (D) Quantification of average FRET yield per cell (*n* = 46; 35; 57). For statistical evaluation, one‐way ANOVA followed by Tukey's test was performed (ns, *p* > 0.05; ****p ≤ 0.0001). (A and D) Standard error of the mean is shown.

Based on results obtained in cotransfection experiments using HEK293T cells, Huang et al. have claimed that human CEACAM1 facilitated surface expression of human TIM‐3 and reported that the majority of CEACAM1‐expressing cells were also TIM‐3‐positive cells [28]. We performed a series of cotransfection experiments in HEK293T cells but could not observe enhanced expression of TIM‐3 upon cotransfection with a vector encoding CEACAM1. Moreover, we could not observe preferential coexpression of TIM‐3 and CEACAM1 in these experiments since in fact, cotransfection with two different expression constructs generally resulted in much higher percentages of double‐positive versus single‐positive cells (Fig. 6A).

Cheung et al. have used B cell and T lymphocyte attenuator (BTLA) and Herpes virus entry mediator (HVEM) fused to fluorescent proteins in conjunction with flow cytometry to demonstrate the in *cis* interaction of these two receptors via Förster resonance energy transfer (FRET) [38]. To use this approach in a study for a potential interaction between CEACAM1 and TIM‐3 in *cis*, we generated expression constructs encoding TIM‐3 and CEACAM1 fused to the fluorescent proteins mRuby3 and mNeonGreen, respectively. They function as a highly efficient FRET donor acceptor pair [39]. HVEM‐mNeonGreen and BTLA‐mRuby3 served as positive controls in these experiments. HEK293T cell were cotransfected with CEACAM1 and TIM‐3 as well as HVEM and BTLA fluorescent fusion proteins. Cells coexpressing BTLA‐mRuby3 and CEACAM1‐mNeonGreen served as a negative control. Flow cytometric analysis revealed that the HEK293T cells expressed the fusion proteins in appropriate levels. Moreover, the occurrence of FRET was observed in cells coexpressing BTLA‐mRuby3 and HVEM‐mNeonGreen as expected. By contrast, we could not detect FRET signals in cells coexpressing TIM‐3‐mRuby3 and CEACAM1‐mNeonGreen (Fig. 6B). We also performed total internal reflection fluorescence microscopy on living cells to measure FRET yields via donor recovery after acceptor photobleaching as this approach supports a highly sensitive and quantitative FRET readout without the need for error prone corrections. To ensure highest possible sensitivity, only cells in the highest decile of acceptor expression were imaged. In line with the results obtained in the flow cytometry‐based assay, FRET signals were readily detected in transient HEK293T cells coexpressing HVEM and BTLA. In contrast, the FRET signals measured in TIM‐3/CEACAM1 coexpressing cells were not different from the background level of the negative control (Fig. 6C and D).

### TIM‐3 signaling inhibits T cells

**Figure 7:**
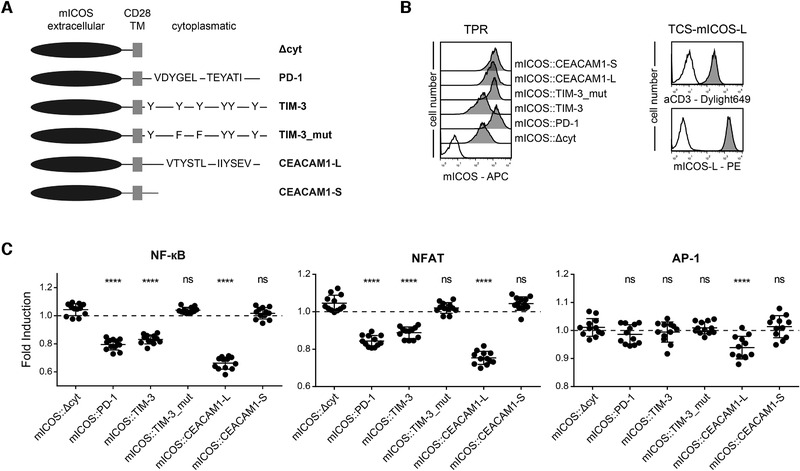
Cytoplasmic sequences of TIM‐3 and CEACAM1 induce inhibitory signals. (A) Schematic of mICOS chimera; (B) Left: surface expression of mICOS::Δcyt, mICOS::PD‐1, mICOS::TIM‐3, mICOS::TIM‐3_mut, mICOS::CEACAM1‐L, and mICOS::CEACAM1‐S on TPR cells (dark gray histograms). Reactivity of mICOS Ab with control TPR is shown as an open histogram. Right: Flow cytometry analysis of TCS‐mICOS‐L. Open histograms: control cells; filled histograms: expression of indicated molecules. (C) mICOS::Δcyt, mICOS::PD‐1, mICOS::TIM‐3, mICOS::TIM‐3_mut, mICOS::CEACAM1‐L, and mICOS::CEACAM1‐S reporter cells were stimulated with control TCS or TCS‐expressing mICOS‐L. For statistical evaluation, one‐way ANOVA followed by Dunnett's test was performed (****p ≤ 0.0001; ns, *p* > 0.05). eGFP, eCFP, and mCherry expression was measured via flow cytometry. Results are shown for four independent experiments performed in triplicates. Reporter activation is shown as fold induction (gMFI of mICOS‐L‐stimulated cells/gMFI of control‐stimulated cells). It should be noted that some data pointes overlap. Control stimulation is shown as a dotted line.

Since our data do not support a role of CEACAM1 as a ligand for TIM‐3, we generated chimeric receptors harboring cytoplasmic sequences derived from human TIM‐3 fused to the extracellular part of a receptor with a known ligand. We assumed that engagement of such chimeric receptors would induce downstream signaling events similar to those induced by engagement of TIM‐3 with its natural ligands. Mouse Inducible T cell costimulator (mICOS), a classical signaling receptor belonging to the Ig‐superfamily, was chosen because of its strong binding to its ligand mICOS‐L. Importantly, ICOS is not expressed on our reporter cells. Expression constructs encoding mICOS::TIM‐3, mICOS::TIM‐3_mut (Y265F and Y272F), or a truncated mICOS molecule lacking cytoplasmic sequences were generated. Chimeric molecules representing the intracellular domain of CEACAM1‐L and CEACAM1‐S were also generated. To confirm the integrity of our strategy, we also generated a construct encoding mICOS fused to the cytoplasmic sequence of PD‐1 a primary coinhibitory receptor on T cells (Fig. 7A). The constructs were expressed in our reporter cells and the resultant reporter cells were probed with an mICOS Ab (Fig. 7B). mICOS‐L was expressed on the TCS to induce signaling of the chimeric receptors during stimulation (Fig. 7C). Engagement of mICOS::PD‐1 constructs by mICOS‐L expressed on our TCS resulted in inhibitory signaling via the cytoplasmic domain of PD‐1, thus validating our approach (Fig. 7D). Stimulation experiments with TIM‐3‐chimera indicated that the cytoplasmic tail of TIM‐3 can mediate inhibitory signaling pathways in the T cell reporter cells because NF‐κB and NFAT activity was strongly reduced upon engagement of mICOS::TIM‐3, whereas a chimera lacking the tyrosine residues corresponding to the Y265 and Y272 in TIM‐3 did not exert inhibitory functions (Fig. 7D). Compared to mICOS::PD‐1, the inhibitory effect of mICOS::TIM‐3 appeared to be weaker but mICOS::TIM‐3 was expressed at lower levels. Interestingly, the cytoplasmic domain of CEACAM1‐L exerted the strongest inhibitory effect on NF‐kB, NFAT, and AP‐1 activation (Fig. 7D). Compared to this strong effect of the chimeric mICOS::CEACAM1‐L, the inhibitory effect of the full‐length CEACAM1‐4L was much weaker (Fig. 2D and Fig. 3B and D). The in *cis* homophilic interaction of CEACAM1 might interfere with in *trans* engagement of CEACAM1‐4L by TCS‐expressed CEACAM1 resulting in a weaker inhibitory effect of full‐length CEACAM1‐4L compared to mICOS::CEACAM1‐L.

Experiments with a CD19‐chimeric antigen‐related receptor fused to cytoplasmic sequences of TIM‐3 also indicate that TIM‐3 can trigger inhibitory pathways in our T cell reporter cells (Supporting information Fig. 3). Taken together, our results suggest that TIM‐3 signaling can inhibit T cells and that tyrosine residues at position 265 and 272 play an important role in this process.

## Discussion

Despite the intense interest in TIM‐3 as a target in cancer therapy, many aspects of the biology of this receptor are still incompletely understood. Human TIM‐3 is upregulated in CD4^+^ and CD8^+^ T cells upon activation and many studies on the functional role of TIM‐3 have focused on T cells. TIM‐3 is often considered as a T cell inhibitory receptor [40], but there are several independently performed studies indicating that TIM‐3 can also have an activating role in T cells [10, 11, 12, 13]. TIM‐3 signaling is considered to depend on the presence of ligands [41], but data linking a particular ligand directly to TIM‐3 functions are scarce.

Huang et al. have reported that CEACAM1 acts as a binding partner for TIM‐3, regulating TIM‐3 mediated tolerance and exhaustion [28]. Here, we have used a T cell reporter system to explore the possibility for an interaction between these receptors. Our data confirmed that CEACAM1 inhibits T cell activation, but we did not obtain any evidence that this receptor has a role in TIM‐3 function. These results prompted us to perform a series of binding experiments to investigate physical interactions between CEACAM1 and TIM‐3. We could not obtain evidence for a specific engagement of these receptors. By contrast, a homophilic interaction of CEACAM1 molecules was readily detected demonstrating the functionality of CEACAM1 proteins used in our study.

Gal‐9 has been reported as a ligand for TIM‐3 and was described to mediate apoptosis of T cells via this receptor [18]. We have previously performed a series of binding experiments and functional studies, and our data did not reveal evidence for a specific interaction between these molecules in the assay systems used in our study [19]. Gal‐9 belongs to a family of growth‐regulatory lectins and selects its counterreceptors in a glycosylation‐dependent manner. Here, we have probed three distinct Gal‐9 preparations with Ig fusion proteins representing TIM‐3 from two different sources and several control fusion proteins. In line with our previous results, we did not obtain any evidence for a specific interaction between Gal‐9 and TIM‐3. We observed that only human Gal‐9 proteins expressed in bacteria strongly bound to different fusion proteins containing the Fc part of human IgG1, whereas Gal‐9 expressed in human cells did not. Additionally, Jurkat cells underwent apoptosis upon treatment with human Gal‐9 expressed in bacteria, while such activities were not detected with Gal‐9 expressed in a human cell line (data not shown). Most functional studies with Gal‐9 have been performed with bacterially expressed proteins and these preparations might have functional properties that are distinct from natural Gal‐9 produced by mammalian host cells. It remains to be clarified, whether, for example, processing by cleavage of the linker between the two lectin domains may underlie this difference, because posttranslational modifications such as glycosylation have so far not been reported for vertebrate galectins that all do not have a signal peptide. The availability of recombinant galectins expressed in human cell lines will allow to determine whether effects observed with galectins expressed in bacteria can be confirmed with protein preparations derived from mammalian expression systems.

Although Huang et al. have reported that CEACAM1 and TIM‐3 are extensively coexpressed [28], in our study, surface CEACAM1 was confined to a highly activated subset of human T cells that have upregulated TIM‐3 in response to prolonged in vitro activation. Furthermore, cotransfection experiments did not indicate that CEACAM1 promotes surface expression of TIM‐3. A negative role of CEACAM1 in T cell responses has been demonstrated by numerous studies and these results were confirmed in the study by Huang et al., which demonstrates T cell hyperresponsiveness and severely impaired tolerance induction in CEACAM1 deficient mice [28]. However, they presented few results that are in support of a functional link between TIM‐3 and CEACAM1. Noteworthy, the crystal structure data of a heterodimer of the V‐domains of CEACAM1 and TIM‐3 presented in their study have since been withdrawn [42]. The results of functional experiments as well as of extensive binding studies presented here do not support the concept of an interaction between CEACAM1 and TIM‐3.

We and others have observed that antibodies targeting human TIM‐3 can enhance human T cell responses when used alone or in combination with PD‐1 blockade [1, 4, 35, 36, 43]. However, this effect of TIM‐3 antibodies was not consistently observed in all studies and appeared to be restricted to certain T cell subsets. TIM‐3 antibodies could act on T cells directly but also indirectly by promoting APC functions. We show that TIM‐3 is constitutively expressed on human monocytes and monocyte‐derived DC. Tolerizing and immune‐potentiating functions have been ascribed to immature and mature DC, respectively [44]. Immature DC express higher levels of TIM‐3 indicating a role of this receptor in promoting immune tolerance via these cells. Although functional human TIM‐3 antibodies are often regarded as antagonists, results obtained by Sabins et al. appear more in line with an agonistic role of TIM‐3 antibodies on human T cells [13]. Gain‐of‐function studies in Jurkat T cells yielded conflicting results, as expression of TIM‐3 appeared to enhance TCR‐dependent signaling pathways [14], whereas other reports described reduced activation of Jurkat cells that were engineered to express TIM‐3 [33, 34]. We have generated several TIM‐3‐expressing Jurkat reporter cell lines to study the impact of TIM‐3 on T cell activation processes. In these experiments, we did not find evidence that the presence of TIM‐3 has a significant effect on T cell activation processes (Fig. 4B; Supporting information Fig. 2 and data not shown). Moreover, we tested the effects of anti‐human TIM‐3 antibodies, previously described to have activity in in vitro stimulation assays with primary human T cells on TIM‐3‐expressing Jurkat reporter cells. However, we did not detect consistently stimulatory effects of these antibodies in our reporter assays (Supporting information Fig. 2 and data not shown). While robust T cell line based reporter cells expressing PD‐1, CTLA‐4, BTLA, TIGIT, or LAG‐3 have been described and can be used to gain mechanistic insights into their function as well as for the evaluation of immune checkpoint inhibitors targeting these receptors, such a system has currently not been described for TIM‐3. This considerably hampers studies on signal transduction processes mediated by this receptor. In our study, we have used chimeric receptors in conjunction with our T cell reporter platform to investigate intracellular signaling via TIM‐3. The results of these experiments demonstrated that the engagement of chimeric receptors harboring the cytoplasmic tail of TIM‐3 profoundly inhibited NF‐κB, NFAT, and AP‐1 activation induced by TCR‐complex signaling. In line with previous studies, our results indicated that tyrosine residues on positions 265 and 272 critically contributed to reporter inhibition mediated by TIM‐3 chimera [34]. Given the contradicting results reported for this receptor, it cannot be excluded that under certain conditions TIM‐3 can also mediate activating signaling into T cells. TIM‐3 is generally thought to function by engaging intracellular signaling pathways in a ligand‐dependent manner [41] and our results are in line with this concept. Based on results presented here and in an earlier study performed by us [19], we propose that TIM‐3 functions independently of CEACAM1 and Gal‐9. The identification of TIM‐3 ligands that mediate regulation of T cell responses via this receptor will help to better understand the biology as well as the therapeutic potential of this receptor.

## Materials and methods

### Sample collection

The study with primary human cells was approved by the ethics committee of the Medical University of Vienna (ECS1183/2016). Procedures with human material were performed in accordance with ethical standards of the ethics committee and the Helsinki Declaration of 1964 and its later amendments. Blood samples were collected from healthy donors. Isolation of PBMCs was performed from heparinized whole blood samples with standard gradient density centrifugation using Lymphoprep^®^ solution (Technoclone, Austria). Generation of immature (iDC) and LPS‐activated monocyte‐derived DC (mature DC) was done as previously described [45].

### Cell culture, antibodies, and flow cytometry

The Jurkat cell line (JE6.1), the human B cell line Raji and the mouse thymoma cell line BW5147 (short designation within this work: BW) were derived from in house stocks. The human monocytic cell line THP‐1 was purchased from ATCC (TIB‐202^TM^). The HEK293T cells used for transfection were provided by A. Carmo, Porto, Portugal. The fluorescent transcriptional TPR‐T cell reporter line, based on the JE6.1 cells has been described previously [31]. TCS, which are based on the BW cell line and express a membrane‐bound human CD3 Ab single chain fragment, have been described in detail [46]. All cell lines used in this study were cultured in RPMI1640 supplemented with 10% FBS, penicillin (100 U/mL), streptomycin (100 μg/mL), and amphotericin B (2.5 μg/mL; all from Sigma Aldrich, St. Louis, MO). For authentication, cells were stained with a panel of antibodies. TPR and T cell stimulators were kept in culture for up to 3 months without perceptible loss of functionality. Cell lines were tested for absence of mycoplasma, using a reporter system described recently [47]. To assess surface expression the following antibodies were used: CD3‐PE‐Cy7 (UCHT1), CD4‐BV421 (RPA‐T4), CD8‐PerCP (HIT8a), CD14‐APC (63D3), CD19‐APC (HIB19), CD25‐PeCy7 (M‐A251), CD66a/CEACAM1‐APC (ASL‐32), CD56‐FITC (HCD56), CD274‐APC (29E.2A3), mICOS‐L‐PE (HK5.3), and isotype control antibodies were purchased from Biolegend (San Diego, CA). TIM‐3‐PE (344823) was from R&D systems (Minneapolis, MN). Surface expression of mICOS::chimera and human ICOS was assessed using h/mICOS‐APC (C398.4A, Biolegend). αCD19 constructs were detected using a biotinylated Strep‐tag II mAb (GenScript, NJ) followed by Streptavidin‐PE staining (BD Pharmingen, San Diego, CA). Surface expression of membrane‐bound anti‐CD3 on TCS was verified using a DyLight‐ 649‐conjugated goat‐anti‐mouse IgG (H + L) Ab (Jackson ImmunoResearch, West Grove, PA). To exclude TCS and Raji cells in reporter assays, they were stained with mCD45.2‐APC (104) or CD19‐APC (HIB19, both Biolegend), respectively. Flow cytometry analysis was performed using FACSCalibur™ or LSRFortessa™ flow cytometers (BD Bioscience, Franklin Lakes, NJ) according to previously published guidelines [48]. FlowJo software (version 10.4.1, Tree Star, Ashland, OR) was used for flow cytometry data analysis.

### CFSE proliferation assay

PBMCs were CFSE‐labeled as described [35]. 1 × 10^5^ cells were stimulated either with SEE (Toxin Technology, Sarasota, FL; final concentration: 100ng/mL) or with immobilized CD3 (OKT3, Ortho Pharmaceutical Corporation, Raritan, NJ; final concentration 1 μg/mL) and CD28 (28.2, Biolegend; final concentration 2 μg/mL) mAbs [49]. After 3, 6, and 10 days of stimulation coexpression of TIM‐3, CD66a/CEACAM1 and CD25 was analyzed on CFSE^low^ CD4^+^ and CD8^+^ T cells. A single data point represents one donor. The full gating strategy is displayed in Supporting information Fig. 1B.

### Retroviral and lentiviral transduction

TCS were modified to express TIM‐3 (UniProt Q8TDQ0‐1), CEACAM1 (CEACAM1‐4S; UniProt P13688‐8), mICOS‐L (UniProt Q9JHJ8‐1), and CD19 (UniProt P15391) via retroviral transduction. Molecules used for transduction of TCS were cloned into the pCJK2 retroviral expression vector [46]. Expression of these molecules was confirmed via flow cytometry.

TPR were engineered to express CEACAM1‐4L (UniProt P13688‐1), CEACAM1‐4S (UniProt P13688‐8), TIM‐3 (UniProt Q8TDQ0‐1), TIM‐3_Δcyt (lacking the cytoplasmic domain), TIM‐3_mut (two tyrosines within the cytoplasmic domain were replaced with phenylalanines; Y265F and Y272F), mICOS::Δcyt, mICOS::CEACAM1‐L, mICOS::TIM‐3, mICOS::TIM‐3_mut, αCD19::Δcyt, αCD19::TIM‐3 via lentiviral transduction followed by puromycin selection (2 μg/mL, Sigma Aldrich). mICOS chimeric constructs consist of a mICOS extracellular domain (aa 1–144 of UniProt Q9WVS0‐1) followed by a codon optimized CD28 transmembrane domain (aa 153–179 of UniProt P10747) and fused to the intracellular domain of TIM‐3 (aa 224–301 of UniProt Q8TDQ0‐1), TIM‐3_mut (aa 224–301 of UniProt Q8TDQ0‐1 with Y265F and Y272F), CEACAM1‐4L (aa 453–526 of UniProt P13688‐1), CEACAM1‐4S (aa 453–464 UniProt P13688‐8), or PD‐1 (aa 192–288 of UniProt Q15116‐1), respectively. mICOS chimera lacking a cytoplasmatic part serves as control (mICOS::Δcyt). A previously described CD19 CAR construct [50], was modified to yield constructs consisting of an extracellular domain containing the GM‐CSF signal sequence, a CD19 scFv (single chain variable fragment), a StrepTag II and a human CD8 hinge sequence followed by a codon optimized CD28 transmembrane domain. The intracellular domain is either truncated (Δcyt) or that of TIM‐3. All constructs transduced into Jurkat cells were cloned into a lentiviral vector pHR‐SIN‐BX‐IRES‐Emerald [51] encoding the puromycin N‐acetyl transferase.

### Reporter assays

Reporter cells (5 × 10^4^ cells/well) were cocultured with TCS (2 × 10^4^ cells/well) or Raji (1 × 10^4^ cells/well) for 24 h at 37°C with 5 % CO_2_. Subsequently, cells were harvested and stained with a mCD45.2 Ab to separate TCS from reporter cells. For staining of Raji cells a CD19 Ab was used. Expression of reporter genes (eGFP, eCFP, mCherry) was then measured via flow cytometry using a LSRFortessa™ flow cytometer equipped with a 561 nm (Yellow‐Green) laser. Geometric mean of fluorescence intensity of viable reporter cells (mCD45.2‐APC negative) was used for further analysis. For some experiments, reporter gene induction in response to stimulation was normalized to either unstimulated or control‐stimulated reporter cells as indicated and expressed as fold induction.

### Binding studies

Flow cytometry based binding studies using Ig fusion proteins were performed with Jurkat cells expressing TIM‐3, CEACAM1 (CEACAM1‐4S) or ICOS. TIM‐3 and CEACAM1 Ig fusion proteins (TIM‐3‐Ig, CEACAM1‐Ig) were purchased from SinoBiological (SB; People's Republic of China). ICOS‐L Ig fusion protein (ICOS‐L‐Ig) was generated and expressed in our laboratory as previously described [49]. Jurkat cells (1 × 10^5^) were incubated with Ig fusion proteins at final concentrations of 31.6, 10, 3.16, 1, and 0.316 μg/mL. Binding was detected via flow cytometry using an APC‐conjugated goat‐anti‐human IgG (Fc‐specific) Ab (Jackson ImmunoResearch). For blocking studies cells were preincubated with a CEACAM1 mAb (clone 283340 mouse IgG_2b_; R&D systems; final concentration of 8 μg/mL). Experiments were performed in duplicates and repeated three times. Geometric mean of fluorescence intensity of viable cells was normalized to background binding of secondary Ab only.

For ELISA‐based binding studies, recombinant human Gal‐9 expressed in *E. coli* or in HEK293T cells and recombinant human CEACAM1 expressed in the murine myeloma cell line NSO cells were purchased from R&D systems. In addition, we used a recombinant human Gal‐9 (Gal‐9 *E. coli* G) purified by affinity chromatography on home‐made lactose‐presenting Sepharose 4B and quality controlled by 1D and 2D gel electrophoresis as described [52, 53]. Proteins were immobilized overnight at 4°C on ELISA plates (Maxisorp; NUNC/Thermofisher Fremont, CA; coating concentration 0.5 μg/mL) described in detail [19]. Ig fusion proteins were added at the indicated concentrations in PBS‐0.5% BSA and incubated for 1 h at room temperature. TIM‐3‐Ig and CEACAM1‐Ig were purchased from SinoBiological (SB); TROP2‐Ig, mouse‐CTLA‐4Ig (mCTLA‐4‐Ig), and another TIM‐3‐Ig were purchased from R&D systems. The plate was washed and HRP‐labeled goat‐anti‐human IgG antibodies (Fc‐specific; Jackson ImmunoResearch) were added and incubated for 1 h at room temperature. Plates were washed and developed using ABTS solution (Roche Applied Science, Mannheim, Germany). Following 20 min incubation, the OD 405 nm was determined using 650 nm as reference wavelength.

For cell–cell binding assays, BW cells were first transduced to express high levels of eGFP or eCFP. Subsequently, CD80 or CEACAM1 were expressed on BW‐eGFP cells, whereas CD28, TIM‐3, or CEACAM1 was expressed on BW‐eCFP cells. Different combinations of BW‐eGFP and BW‐eCFP cells (1 × 10^5^ cells each) were mixed in 200 μl full RPMI medium in 1.2 mL micro titer tubes (Biozym Scientific, Hessisch‐Oldendorf, Germany). Following an incubation step at 37°C with 5% CO_2_ for 1 h, cells were gently mixed and cell‐conjugate formation was determined by flow cytometry.

### FRET analysis via flow cytometry

For analysis of in *cis* interaction, expression constructs encoding human TIM‐3 or BTLA fused with mRuby3, and CEACAM1‐4L or HVEM fused with mNeonGreen were used. HEK293T cells were transiently transfected to express CEACAM1‐mNeonGreen, TIM‐3‐mRuby3, BTLA‐mRuby3, or HVEM‐mNeonGreen. Additionally, HEK293T cells that coexpress CEACAM1‐mNeonGreen and TIM‐3‐mRuby3, BTLA‐mRuby3 and HVEM‐mNeonGreen or CEACAM1‐mNeonGreen and BTLA‐mRuby3 were generated. mNeonGreen was excited with 488 nm laser light and emission was detected using FITC filters. Laser light (561 nm) was used to excite mRuby3 and emission was measured with PE filter settings. For measuring the FRET signal 488 nm excitation and a 600 nm longpass filter for emission were used.

### Microscopy

HEK293T transfected with fluorescent fusion proteins as mentioned above were cultured overnight in LabTek chambers coated with 10 μg/mL fibronectin in PBS for 2 h at 37°C. Image acquisition was performed on a T*i*‐E inverted microscope (Nikon, Tokyo, Japan) equipped with a 100× objective (SR Apo TIRF, Nikon) and an Andor iXon Ultra‐897 EM‐CCD camera (Andor Technologies, Belfast, UK). The 488 nm (mNeonGreen) and 561 nm (mRuby3) laser line were used for fluorophore excitation in an objective‐based total internal reflection configuration and emitted light was split on the camera using a Optosplit II (Cairn Research, Faversham, UK) equipped with a 561 nm dichroic mirror, 525/50 (mNeonGreen) and 575LP (mRuby3) filters (Chroma, Bellow Falls, VT). MetaMorph imaging software (Molecular Devices, Downingtown, PA) was used to control the devices. Image analysis was performed using ImageJ (Version 1.51, National Institute of Health, Washington, DC; [54]).To determine FRET efficiencies, donor emission before (pre) and after acceptor photobleaching (post) as well as background signal was recorded and measured for a region centrally located in the laser beam. Complete ablation of the acceptor was confirmed, and FRET yields were calculated either pixelwise or for each individual region using the following formula:

$$\mathrm{FRET}\;=\frac{\mathrm{donor}\left(\mathrm{post}\right)-\mathrm{donor}\left(\mathrm{pre}\right)}{\mathrm{donor}\left(\mathrm{post}\right)-\mathrm{background}}.$$

### Statistics

For experiments with CEACAM1 and CEACAM1/TIM‐3 reporter cells and primary cells (Fig. 1A–C and E), two‐tailed paired *t*‐test was performed. For experiments with reporter cells expressing TIM‐3, mICOS::CEACAM1‐L or aCD19::TIM‐3, a two‐tailed unpaired *t*‐test was performed. Statistical analysis of TIM‐3 expression on DC, mICOS::TIM‐3 reporter experiments as well as for FRET experiments were performed using one‐way ANOVA followed by Tukey's multiple comparison test. Statistical calculations were performed using GraphPad Prism (levels of significance were categorized as follows: ns, not significant; *p ≤ 0.05; **p ≤ 0.01; ***p ≤ 0.001; ****p ≤ 0.0001).

## Author contributions

The conceptual design of this work was done by ADSL, JL, and PS. The methodology was developed by ADSL, SJ, FK, JH, JL, and PS. The acquisition of data was done by ADSL, JL, and FK. The analysis and interpretation of data were done by ADSL, FK, JH, JL, and PS. GJZ and HJG provided resources and important reagents for this work. This manuscript was written by ADSL, JL, and PS. Review and revision of the manuscript were done by all authors.

## Conflict of interest

GJZ reports personal fees from Alexion, Bristol‐Myers Squibb, MedAhead, the Austrian Chamber of Physicians, Pfizer, UCB Pharma, Merck Sharp & Dohme, GlaxoSmithKline, and AbbVie outside the submitted work. PS reports personal fees from Bristol‐Myers Squibb outside the submitted work. All other authors declare no financial or commercial conflict of interest.

**Abbreviations**
BTLA: B and T lymphocyte attenuator
CEACAM1: carcinoembryonic antigen‐related cell adhesion molecule
FRET: Förster resonance energy transfer
Gal‐9: galectin‐9
HEK: human embryonic kidney
HVEM: Herpes virus entry mediator
mICOS: murine Inducible T cell costimulator
SEE: staphylococcal enterotoxin E
TCS: T‐cell stimulator cells
TPR: triple parameter reporter

## Supporting information

Supporting Information
