# Supplementary material for: TIM‐3 and CEACAM1 do not interact in cis and in trans
Source: Eur J Immunol. 2020 Apr 28;50(8):1126–41. doi: 10.1002/eji.201948400 (PMC7496933; doi:10.1002/eji.201948400)
Supplement: Supplementary file 1 — Supporting Information [file EJI-50-1126-s001.pdf]

Supplementary information

TIM-3 and CEACAM1 do not interact *in cis* and *in trans*

Annika De Sousa Linhares[1], Florian Kellner[2], Sabrina Jutz[1], Gerhard J. Zlabinger[3], Hans-Joachim Gabius[4], Johannes B. Huppa[2], Judith Leitner[1*] and Peter Steinberger[1,5*]

[1]Division of Immune Receptors and T Cell Activation, Center for Pathophysiology, Infectiology, Medical University of Vienna, Vienna, Austria

[2]Department of Molecular Immunology, Immune Recognition Unit, Institute for Hygiene and Applied Immunology, Center for Pathophysiology, Infectiology and Immunology, Medical University of Vienna, Vienna, Austria

[3]Division of Clinical and Experimental Immunology, Center for Pathophysiology, Infectiology, and Immunology, Institute of Immunology, Medical University of Vienna, Vienna, Austria

[4]Institute for Physiological Chemistry, Faculty of Veterinary Medicine, Ludwig-Maximilians-University Munich, Munich, Germany

[5]Lead contact

*****contributed equally; correspondence to: judith.a.leitner@meduniwien.ac.at (Judith Leitner) or peter.steinberger@meduniwien.ac.at (Peter Steinberger)

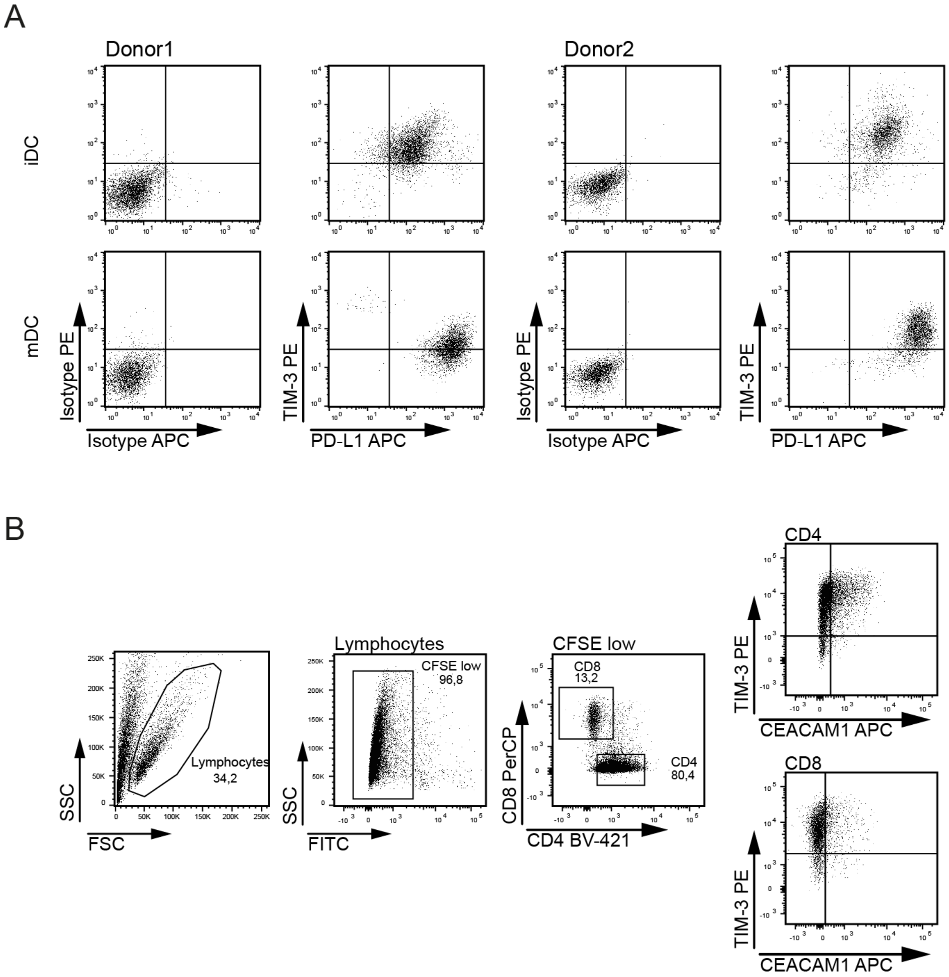

Suppl.Fig. 1: *TIM-3/CEACAM-1 expression on primary cells*

A) Downregulation of TIM-3 during DC maturation. Expression of TIM-3 and PDL1 on immature (upper panel) and mature (lower panel) human DC are shown. Left: Isotype control staining, right: expression of PDL1 and TIM3. Dot plots of two representative donors are shown. B) Gating strategy to Fig. 1E is shown. Viable cells were identified by their forward/side scatter properties. In stimulated samples the gate was set on the CFSE^{low} population. Subsequently, CD4 and CD8 subsets were gated and analyzed for TIM-3 and CEACAM-1 expression.

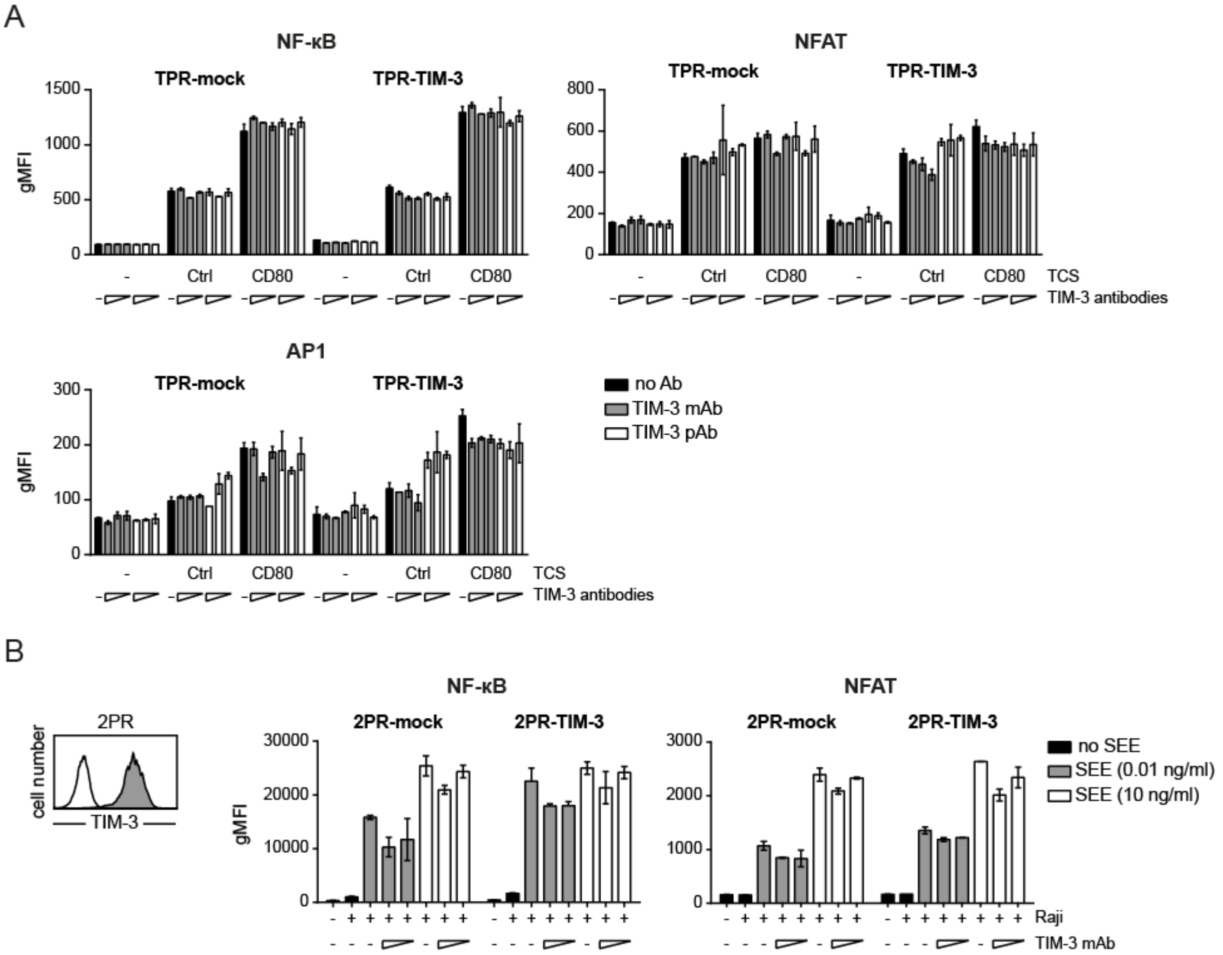

Suppl. Fig. 2: *Evaluation of TIM-3 antibodies*

(A) Control reporter cells and triple parameter reporter cells expressing TIM-3 (TIM-3; expression of TIM-3 is shown in Fig. 4A) were left unstimulated or stimulated with TCS expressing no co-stimulatory molecules (TCS-Ctrl) or with TCS expressing human CD80. TIM-3 antibodies were added at final concentrations of 10, 3.16 or 1 µg/ml. Following 24 h of co-culture reporter gene expression (eGFP, eCFP and mCherry) was measured via flow cytometry. **(B)** Left: Flow cytometry analysis of 2PR reporter cells (JE6.1 dual parameter reporter; NF-κB::eGFP; NFAT::eCFP) expressing TIM-3 (grey histogram). Reactivity of TIM-3 antibodies to control 2PR is shown in the open histogram. Middle and Right: Control 2PR and 2PR expressing TIM-3 ($5x10^4$ cells/well) were left unstimulated or co-cultured with Raji cells ($1x10^4$ cells/well) Staphylococcal Enterotoxin E (SEE; Toxin Technology, Inc; Sarasota, FL) was added as indicated. TIM-3 mAb was added at final concentrations of 5 or 1 µg/ml. Following 24 h of co-culture reporter gene expression was measured via flow cytometry. **(A,B)** TIM-3 mAb (#344823; Rat IgG2a) and TIM-3 pAb (polyclonal goat IgG antigen-affinity purified antibody) were purchased from R&D systems. Standard deviation is shown.

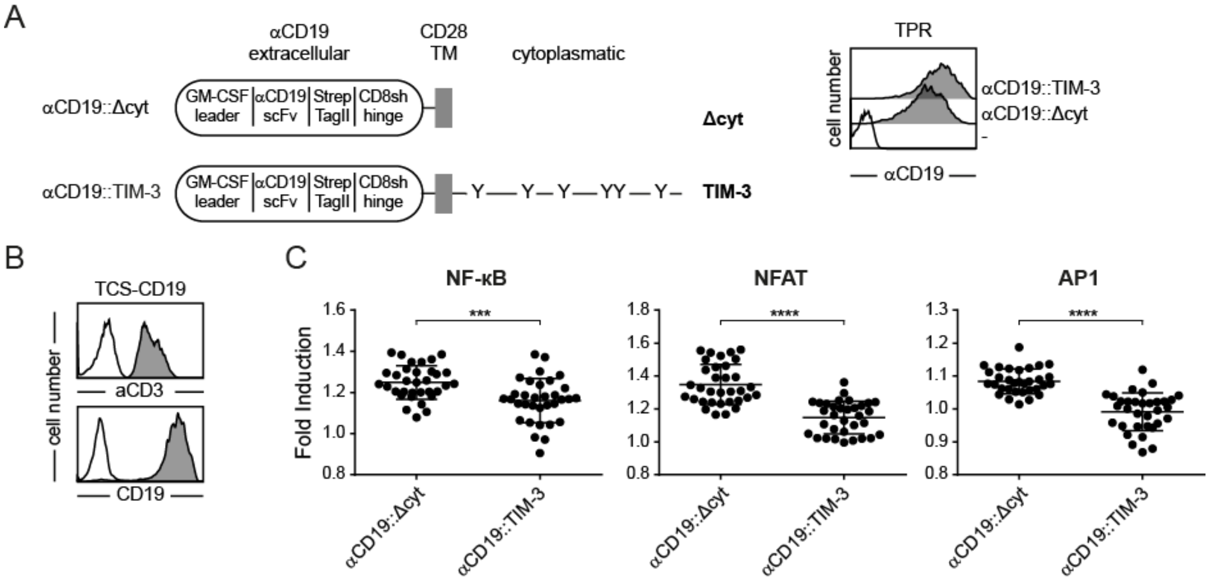

Suppl. Fig. 3: Evaluation *aCD19::TIM-3 chimeric molecules*

(A) Left: Schematic of αCD19 chimera. Right: Surface expression of αCD19::Δcyt and αCD19::TIM-3 on TPR cells. αCD19 constructs were detected using a Strep-tag II-biotin antibody (grey histogram). Reactivity of antibodies to control TPR is shown in the open histogram. **(B)** Expression of membrane-bound anti-CD3 antibody and CD19 on TCS-CD19. Reactivity of the respective antibodies to control cells is shown as open histograms **(C)** Triple parameter reporter cells expressing αCD19:: Δcyt and αCD19::TIM-3 were stimulated with TCS expressing human CD19 or control-TCS. Following 24 h of co-culture reporter gene expression (eGFP, eCFP and mCherry) was measured via flow cytometry. Results are shown for eleven independent experiments performed in triplicates. Reporter activation is shown as fold induction (gMFI of TCS-CD19 stimulated reporter cells/gMFI of control-TCS stimulated reporter cells). Unpaired t-tests were used for statistical analysis (***p ≤ 0.001; ****p ≤ 0.0001).
